# A250 CYTOKINE MULTI-OMICS AND MACHINE LEARNING IDENTIFY MIP1ALPHA AS A NOVEL MEDIATOR IN INFLAMMATORY BOWEL DISEASE

**DOI:** 10.1093/jcag/gwad061.250

**Published:** 2024-02-14

**Authors:** E M O'Brien, C Potdar, D Mulder

**Affiliations:** Biomedical and Molecular Sciences, Queen's University, Kingston, ON, Canada; Biomedical and Molecular Sciences, Queen's University, Kingston, ON, Canada; Pediatrics, Queen's University, Kingston, ON, Canada

## Abstract

**Background:**

Macrophage inflammatory protein 1 alpha (MIP1a) is a proinflammatory cytokine previously related to murine models of inflammatory bowel disease (IBD), but not definitively in the human disease. MIP1a acts as a chemoattractant of immune cells from the blood to the gut mucosa. We aimed to delineate the alterations to MIP1a and macrophages in relation to a range of IBD severity.

**Aims:**

To characterize the changes to MIP1a production and localization in response to disease activity in IBD.

**Methods:**

Both IBD (n=63) and control patients (n=118) were enrolled in this study (HSREB 6033229). Cytokine profiles were investigated using a 17-plex multi-fluorescent bead-based immunoassay (FirePlex, Abcam, Cambridge, UK) on serum from a subset of patients. Disease activity and macrophage levels were extracted from the clinical record. Activity was quantified using the Physician Global Assessment Score. Machine learning (ML) was performed with custom R scripts utilizing the tidymodels package (version 1.1) to determine the optimal model. Immunohistochemistry (IHC) was used to localize MIP1a in patient biopsies and quantified using QuPath software (v0.4).

**Results:**

An extreme gradient boost ML model was found to have optimal sensitivity and specificity for predicting disease activity based on serum cytokine levels. Within this model lower levels of serum MIP1a were associated with higher severity of IBD activity. However, in GI mucosal biopsies, the percentage of MIP1a positive cells in colon tissue increased with the severity of IBD. Macrophage concentrations in the peripheral blood were higher in patients on prednisone, and relatively similar across all other medications used in our cohort.

**Conclusions:**

With the data collected, we identify MIP1a as a possible prognostic tool for quantifying IBD activity.

**Funding Agencies:**

CIHR

